# Positive Selection of Specific Antibodies Produced against Fusion Proteins

**DOI:** 10.3390/mps3020037

**Published:** 2020-05-08

**Authors:** Lukas Kramberger-Kaplan, Tina Austerlitz, Holger Bohlmann

**Affiliations:** Department of Crop Sciences, Institute of Plant Protection, University of Natural Resources and Life Sciences, 1180 Vienna, Austria; lukaskramberger@hotmail.com (L.K.-K.); tina.austerlitz@boku.ac.at (T.A.)

**Keywords:** *E. coli*, Thioredoxin, GFP, FLAG-tag, His-tag, CusF

## Abstract

A method for the positive selection of specific antibodies for target proteins expressed as fusion proteins for the production of antiserum is presented. As proof of concept, the fusion protein FLAG::His::GFP::His::FLAG was expressed in *Escherichia coli*, purified, and used for the immunization of rabbits. The obtained serum was precleared via protein A affinity. A CusF::FLAG fusion protein was expressed in the periplasm of *E. coli* and purified. GFP without tags was also expressed in *E. coli* and purified via organic extraction. These proteins were then coupled to NHS-activated sepharose and used for the positive selection of Anti-GFP and Anti-FLAG antibodies. The obtained sera were tested for their specificity against different protein samples and fusion proteins in Western blots. A high specificity of the antibodies could be achieved by a single affinity chromatography step. In general, we advise to express the target protein with different tags and in different *E. coli* compartments for antibody production and affinity chromatography.

## 1. Introduction

Antibodies are important tools in many areas of molecular biology research but are also increasingly used in medicine [1]. They can be produced by immunizing rabbits or other mammals or hens. Antibodies that are used in medicine can also be produced in *Escherichia coli* or other expression systems, including plants, after cloning the genes and establishing corresponding libraries [2,3]. Polyclonal antibodies contain a mixture of antibodies that are directed against different epitopes of the antigen, while monoclonal antibodies can be selected to contain only one epitope-specific antibody [4]. In many cases, polyclonal antibodies are more than sufficient and are mostly produced in rabbits [5]. One problem in obtaining antibodies is often the production of the antigen that is needed for immunization. This can be the case if the antigen, usually a protein, is only expressed at very low levels. Purifying such proteins can be very time consuming, requiring months or even years of work. In such cases the protein is often expressed in an expression system such as *E. coli*, yeast, or *Pichia pastoris* and purified. However, even then, the target protein might be difficult to obtain. 

To circumvent problems of solubility or expression, target proteins are produced as fusion proteins [6]. These fusions, i.e., His-tag, maltose binding protein, thioredoxin, etc., can increase the solubility of the protein but may also be used in affinity purification of the fusion protein. For instance, His-tag-containing proteins can be purified with metal affinity chromatography [7]. Fusion proteins can then be used as antigens for immunization (for example, in rabbits). The antibodies thus obtained will then be a mixture of antibodies directed against the target protein and against the fusion partner. If the fusion protein was produced in *E. coli*, there will also be antibodies against *E. coli* proteins, because the fusion proteins that are used as antigens are not 100% pure. It will therefore be necessary to purify the antibodies from those against the fusion partners and the *E. coli*–specific antibodies. 

A common method to purify antibodies is negative selection [8]. This method uses protein extracts from *E. coli* expressing only the empty vector (without the target protein). The cells are lysed by sonication, and the supernatant is immobilized on a nitrocellulose membrane. The membrane is then incubated with the corresponding polyclonal antiserum in order to remove unspecific antibodies.

In our lab, we are mostly using a His-tag-containing thioredoxin as a fusion for antimicrobial peptides in the cytoplasm of *E. coli* because it was previously found to be the most promising partner for the expression of viscotoxin in *E. coli* [9]. We used these fusion proteins to produce polyclonal antibodies in rabbits. However, we found that most of the antibodies was directed against the His-tag-thioredoxin and not against our target protein. Using negative selection to purify these antibodies was not very effective (data not shown). We have therefore established a positive selection scheme using the protein of interest coupled to a different fusion partner. In addition, we also expressed this fusion in a different compartment—the periplasm. Using this fusion protein in affinity chromatography yielded very pure antibodies against our target protein.

## 2. Methods

### 2.1. Cloning Procedures

**Figure 1:**
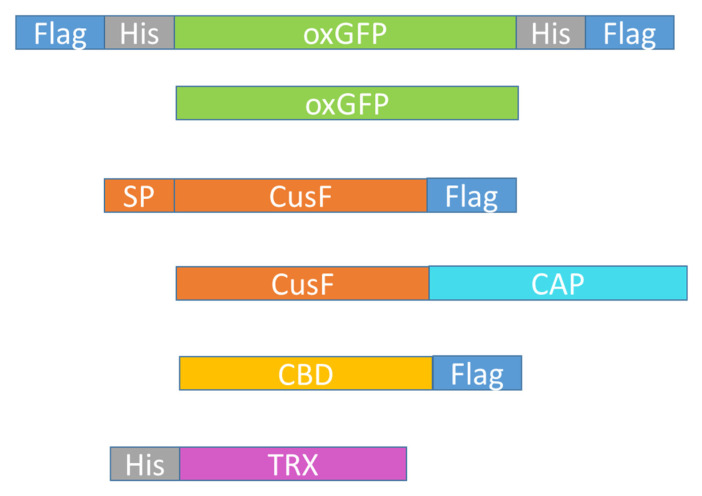
Constructs used for the expression of proteins.

All constructs were cloned into a pET vector [9] which was modified to include a NdeI cloning site at the start codon in addition to the BamHI site behind the stop codon. The different proteins that were used are shown in Figure 1. The antigen construct was produced by amplifying oxGFP (oxidizing environment-optimized green fluorescent protein; from now on we will refer to this only as GFP) with primers (Table S1) oxGFPHisFlagBamrev and FlagHisoxGFPNdefor, thereby introducing His and FLAG tags at both sides of GFP (Figure S1). The PCR (polymerase chain reaction) product was digested with NdeI and BamHI and ligated to the pET vector digested with the same restriction enzymes. Primers sfGFPforNde and FLOURrevBam were used to amplify GFP without tags (Figure S2).

A CusF::FLAG construct was cloned by first amplifying CusF from *E. coli* using primers CusFSPforNde and CusFTEVrevBam. This PCR produced the CusF protein (including the signal peptide) with a C-terminal GS_3_ linker followed by a TEV site. This construct was cloned as previously described. It was then used as the template in a second PCR to attach a FLAG-tag to the linker using the primer GS3FLAGrevBam (Figure S3).

CusF::CAP (Figure S4) was cloned by first producing CusF (including the signal peptide) with a C-terminal GS_3_ linker followed by a TEV (tobacco etch virus) site as previously described. CAP was amplified with primers TEVCAPfor and CAPrevBam from Arabidopsis DNA. Both parts were then fused together by overlap PCR and primers CusFSPforNde and CAPrevBam.

A CBD::FLAG (Figure S5) construct was amplified with primers CBDCEXforNde and GS3FLAGrevBam. It contains the Cex sequence from a beta-1,4-glycanase from the bacterium *Cellulomonas fimi*.

A His::TRX (Figure S6) protein was obtained by TEV digestion of a fusion protein containing an antimicrobial peptide. It still contains the TEV site at the C-terminus.

### 2.2. Expression and Purification of FLAG::His::GFP::His::FLAG

A 1 l culture of the GFP construct in BL21 was grown at 37 °C until it reached an OD_600_ of 0.8 and was then induced with 1mM IPTG. Expression was done o/n at 30 °C. Cells were then centrifuged for 30 min at 4000 rpm and the pellet was dissolved in 35 mL of His-tag binding buffer (see below). The cell suspension was sonicated for 2 min and the cell lysate was centrifuged at 18000 rpm at 4 °C for 30 min, and the supernatant was immediately transferred to a 50 mL tube and kept in the fridge at 4 °C until purification.

The GFP fusion was purified using an Äkta10 FPLC (GE Healthcare, Vienna, Austria) system. Buffer conditions were 20 mM sodium phosphate, 0.5 M NaCl, pH 7.4 containing 50 mM imidazole for binding and 20 mM sodium phosphate, and 0.5 M NaCl, pH 7.4 with 500 mM imidazole for elution. The protein lysate was loaded onto a HisTrap HP column (GE Healthcare, Vienna, Austria), washed with 5–10 CV of binding buffer and eluted with 100% elution buffer without a gradient. The proteins were then precipitated with acetone and dissolved in 35 mL of anion exchange binding buffer (20 mM sodium phosphate, pH 8). The protein solution was loaded onto a HiTrap Q HP column, washed with 5–10 CV of binding buffer and then eluted with a gradient from 0%–100% elution buffer (20 mM sodium phosphate, 1 M NaCl, pH 8) over 30 min. Detection was done with 280 nm and 488 nm. Then, 1 mg of the purified GFP fusion protein was used for antibody production in rabbits. 

### 2.3. Antibody Production

The antibody was produced in rabbits by BioScience (Göttingen, Germany) according to the following protocol. The first injection used 250 µg antigen (FLAG::His::GFP::His::FLAG) in 500 µL with 500 µL Freund’s complete adjuvant. The first boost was done at day 21, using 250 µg antigen in 500 µL, but this time with incomplete Freund’s adjuvant. First blood was taken at day 35, and the second boost (as first boost) was done at day 49. Second blood was taken at day 53. Third boost (as first boost) was done at day 67. Final bleeding was at day 91. For the experiments, we used the antibodies from the final bleeding.

### 2.4. Expression and Purification of GFP

Expression of GFP without tags was done as above. However, the first purification step was an anion exchange chromatography as described above for the GFP fusion. Next, an organic extraction was done as previously described in Yakhnin et al. [10] with a slightly modified protocol. Solid ammonium sulfate was added to the anion exchange eluate to a final concentration of 2.8 M. The suspension was then extracted with 0.5 volumes of ethanol through vigorous shaking, and the phases were separated by centrifugation at 4000 rpm at RT for 5 min. GFP extraction could be observed by the bright green fluorescence of the upper phase in daylight. The GFP-containing phase was collected, and 1 volume of chloroform was added. GFP extraction was performed as before by shaking and centrifugation.

### 2.5. Purification of Periplasmic CusF::FLAG

BL21 cells were grown in 800 mL of TB medium containing 50 µg/mL of kanamycin and induced with 1 mM IPTG at an OD_600_ of 0.55. The periplasmic fraction was obtained as follows [11]. After 18 h at 20 °C, the cells were centrifuged at 4000 rpm for 20 min at 4 °C. The pellet of 18.5 g bacteria was then re-suspended in 800 mL of precooled 30 mM Tris/HCl, 20% sucrose, pH 8.0. Under continuous agitation 500 mM EDTA was added dropwise to a final concentration of 1 mM. The cells were kept in the sucrose solution on ice for a total of 15 min.

The cells were then centrifuged at 4000 rpm for 30 min at 4 °C. The pellet was re-suspended in 150 mL of icecold 5 mm MgSO_4_ and incubated on ice under mild agitation for 15 min. Finally, the supernatant was separated from the cells by centrifugation for 40 min at 15000 rpm at 4 °C and then dialyzed against 20 mM Na_2_HPO_4_, 400 mM NaCl, pH 7.4.

The CusF protein was purified from the periplasmic fraction via copper ion affinity chromatography using a HisTrap HP column (GE Healthcare, Vienna, Austria). The column was previously strapped and reloaded with copper ions [12]. Elution was done with 500 mM imidazole, 20 mM Na_2_HPO_4_, 400 mM NaCl, pH 7.4, and 6 mg protein were obtained. After dialysis against ddH_2_O, 0.065% TFA proteins were separated via RPC in batches of 1 mg. The large peaks around 40% ACN were collected and verified by PAGE. A total of 5 mg CusF::FLAG was obtained.

### 2.6. Purification of Antibodies

Antibodies were purified from serum using HiTrap ProteinA HP 1 mL columns (GE Healthcare, Vienna, Austria). The serum was centrifuged for 30 min at 4 °C with 15,000 rpm and filtered through a 0.45 µm syringe filter. After dialysis against 20 mM Na_2_HPO_4_, pH 7.4, the serum was applied to the proteinA column in batches of 5 mL. Elution was done with 100 mM citric acid, pH 4.0 without gradient. The recovered eluates were immediately treated with 10% (v/v) 1 M Tris pH 9.0 to neutralize pH and stabilize the antibodies. The eluted fractions were combined and dialyzed against 75 mM Tris, pH 8.0 including 0.1% (w/v) NaN_3_ and kept at 4 °C for short-term storage. 

### 2.7. Coupling of Proteins to NHS-Activated Sepharose 

Proteins were coupled to HiTrap™ NHS (N-hydroxysuccinimide)-activated HP 1 mL columns (GE Healthcare, Vienna, Austria) according to the manufacturer‘s instructions. For that, 4 mg of CusF::FLAG or 4 mg of GFP were suspended in one CV of coupling buffer (0.2 M NaHCO_3_, 0.5 M NaCl pH 8.3) and allowed to solubilize for 1 h at room temperature. The column was prepared by acidification with 6 CV of ice-cold 1 mM HCl. The ligand was injected into the column directly after acidification. With two syringes on each end of the column, the ligand solution was repeatedly pumped forth and back through the column over a period of 4 h at room temperature. Then, the column was washed and deactivated by repeatedly applying 6 CV of buffer A (0.5 M ethanolamine, 0.5 M NaCl, pH 8.3). This was followed by 6 CV of buffer B (0.1 M acetate, 0.5 M NaCl, pH 4.0) and back to buffer A with an incubation period of 30 min at room temperature. Afterward, the washing and deactivation was repeated in the opposite order (B, A, B). Finally, 20 mM Tris pH 7.0 was injected to stabilize the pH at neutral. For storage the columns were kept in 50 mM Na_2_HPO_4_, 0.1% (w/v) NaN_3_, pH 7.0. 

The protein A purified antibodies in 75 mM Tris, pH 8.0 were pumped through the antigen coupled NHS-columns in batches of 3–5 mL with a flowrate of 0.2–1 mL/min using the P-900 sample pump. After binding, the column was washed with binding buffer and then bound antibodies were eluted with 3–5 CV of elution buffer (500 mM NaCl, 100 mM glycine, pH 2.7). Again, the eluates were immediately neutralized. After the purification and concentration of the antibodies via repeated application to the protein A column the collected fractions were again neutralized, combined and dialyzed against PBS buffer. After dialysis 50% (v/v) of glycerol and 0.01% (w/v) NaN_3_ were added, and the solutions stored at −20 °C.

### 2.8. Western Blot Analysis

C3030, antigen (FLAG::His::GFP::His::FLAG), CusF::CAP and CBD::FLAG were grown in 5 mL cultures at 37 °C. C3030 was not induced with IPTG. The other probes were induced with 1 mM IPTG and grown at 30°C. 1 mL of each culture was centrifuged in 1.5 mL Eppendorf tubes at 13,000 rpm, 4 °C in a tabletop centrifuge. The pellet was dissolved in 1 mL of SDS-PAGE loading buffer containing 100 mM DTT, heated to 95 °C for 10 min, centrifuged full speed for 10 min and directly taken for SDS-PAGE. Purified proteins for SDS-PAGE were treated the same way. The His::TRX used was a byproduct of previous protein expressions and was obtained after TEV digestion. Purification of GFP and CusF::FLAG proteins has been described above. Purified proteins for SDS-PAGE were treated as described above.

After SDS-PAGE the proteins were transferred to a 0.45 µm pore size nitrocellulose membrane (Amersham™Protran™, GE Healthcare, Vienna, Austria) using a Trans Blot^®^ SD Semi-Dry Transfer Cell (BIO-RAD, Vienna, Austria) at 18 V for 20 min. The membrane was incubated in PBS with 2.5% skim milk powder and 0.1% Tween20 with antibodies diluted to 1:3000 for one hour at room temperature. After washing, the secondary antibody IRDye^®^800CW Donkey Anti Rabbit (LI-COR Biosciences GmbH, Bad Homburg, Germany) was added in a dilution of 1:15,000 in PBS containing 0.1% Tween20. Incubation was done for 30 min at room temperature and slightly shaking. After washing and drying, the membranes were analyzed at 700 nm and 800 nm in an Odyssey infrared scanner (LI-COR Biosciences GmbH, Bad Homburg, Germany).

## 3. Results

We needed antibodies against GFP and against the FLAG-tag. Since the FLAG-tag is only eight amino acids long [13], it is not possible to use it directly for immunization. Instead of coupling it to a different carrier, we produced a construct fusing it to GFP. To be able to purify such a protein, we also included a His-tag that would allow us to use affinity chromatography. In order to increase the amount of antibodies against the FLAG-tag, we added a FLAG-tag at the N-terminus and at the C-terminus (Figure 1). The His-tag was included between both FLAG-tags and GFP. This fusion protein (FLAG::His::GFP::His::FLAG) was expressed in *E. coli* and purified. It was used to produce a polyclonal antibody in rabbit.

**Figure 2:**
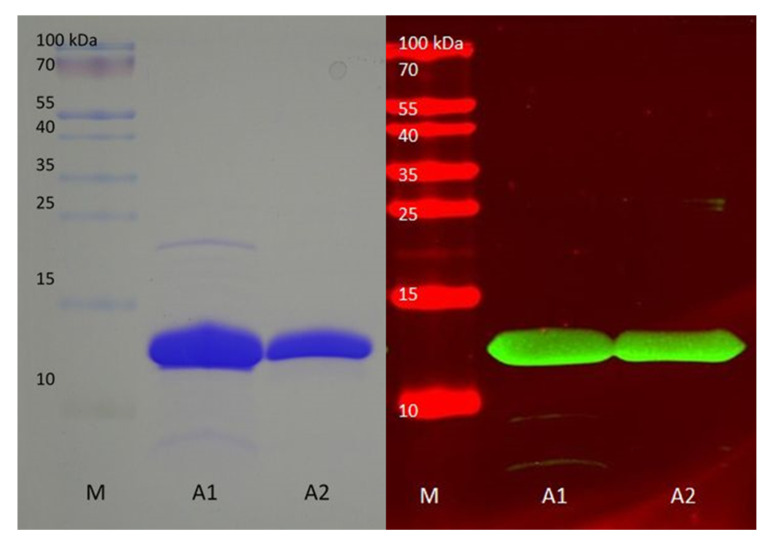
CusF purified from the *Escherichia coli* periplasm. **Left**, coomassie stained SDS-PAGE (sodium dodecyl sulfate polyacrylamide gel electrophoresis) gel; **Right**, Western blot with anti-FLAG antibody. A1 and A2 are fractions from reverse phase chromatography after copper ion affinity chromatography. Lane A1 shows minor contaminations. Lane A2 represents the second half of the peak that delivered higher purity of the CusF::FLAG protein.

In order to purify specific anti-FLAG and anti-GFP antibodies from the mixture of antibodies, we bound respective corresponding proteins (Figure 1) to NHS columns. For the isolation of anti-GFP antibodies we purified GFP with different chromatographic steps as described in methods. Anti-FLAG antibodies were isolated with a CusF::FLAG fusion protein expressed in the periplasm. CusF is a copper binding protein from *E. coli* that is secreted to the periplasm and can be isolated using immobilized metal ion affinity chromatography with copper ions [12,14]. Pure CusF::FLAG fusion protein could be isolated in a large amount from the *E. coli* periplasm (Figure 2). 

**Figure 3:**
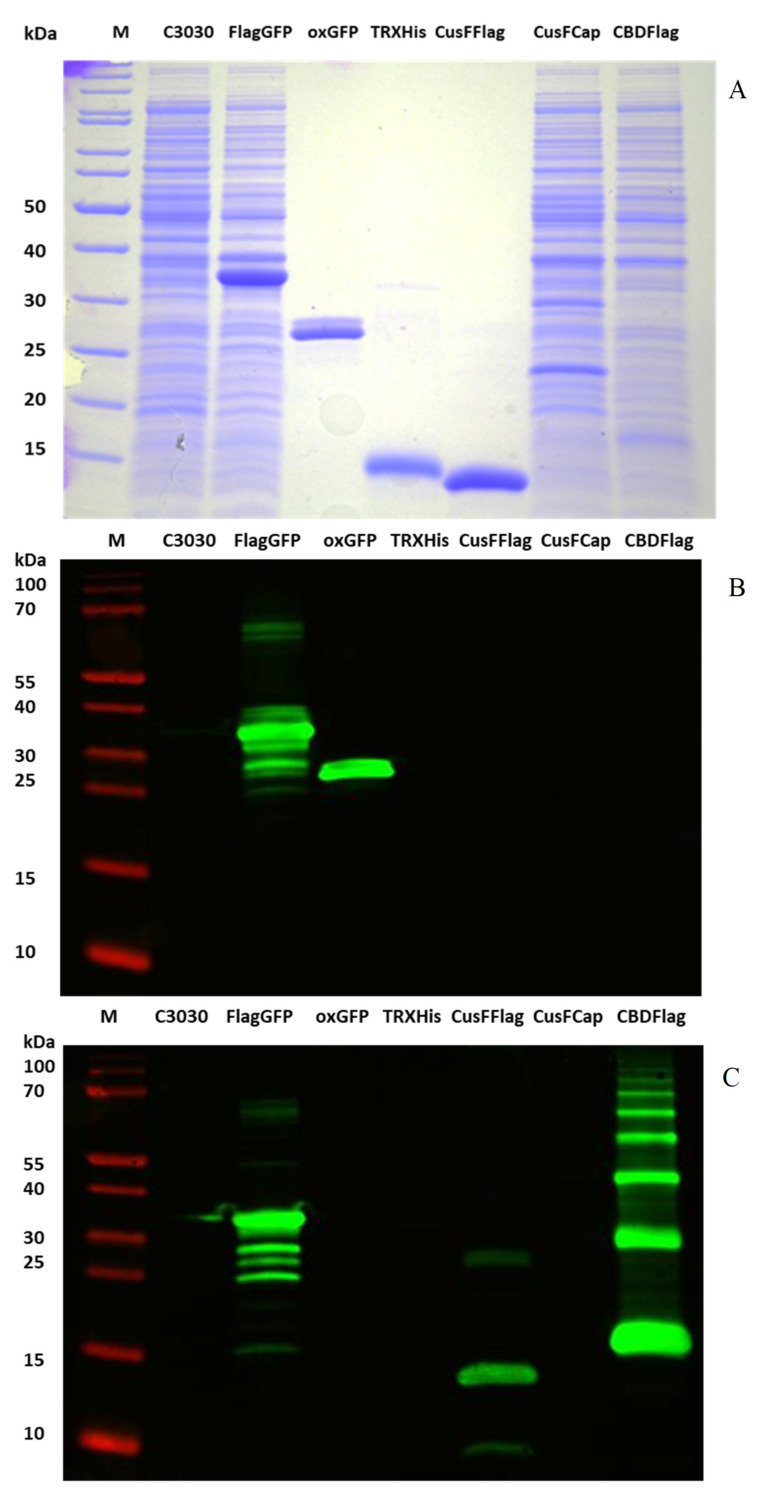
Coomassie stained SDS PAGE gel (**A**) containing total protein from *E. coli* (C3030) without vector and extracts after expression of the antigen construct (FLAG::His::GFP::His::FLAG), CusF::CAP and CBD::FLAG. Purified proteins that were loaded included GFP, His::TRX and CusF::FLAG. (**B**), Western blot with anti-GFP antibody; (**C**), Western blot with anti-FLAG antibody.

The Anti-GFP and Anti-FLAG antibodies were tested on Western blots against different purified proteins and *E. coli* extracts for their specificity (Figure 3). As a control we used uninduced *E. coli* cells (C3030). In another lane we added the extract from induced *E. coli* expressing the GFP fusion construct (FLAG::His::GFP::His::FLAG) that was used for antibody production. In addition, we used the purified GFP that was utilized to isolate anti-GFP antibodies. The His-TRX protein was a byproduct of fusion protein digestion and purification of an antimicrobial peptide (data not shown). Furthermore, the purified CusF::FLAG protein from the periplasm that was used for the isolation of anti-FLAG antibodies was run on the gel. Finally, extracts from induced *E. coli* expressing a CusF::CAP and a CBD::FLAG fusion, respectively, were included. Figure 3A shows the coomassie stained gel. Western blots corresponding to this gel were reacted with anti-GFP antibodies (Figure 3B) and anti-FLAG antibodies (Figure 3C), respectively. The anti-GFP antibody recognized only the purified GFP and the GFP fusion protein in *E. coli* extract. There was no cross reaction with any of the other proteins. Contrary, the anti-FLAG antibody did not react with the purified GFP protein. It did however recognize the GFP fusion protein which contains FLAG-tags. It also recognized the purified CusF::FLAG fusion and the FLAG-tag of the CBD::FLAG fusion protein (this protein runs as multimers on the gel resulting in several bands). There was no reaction with the His::TRX protein. Thus, from a mixture of antibodies, we isolated anti-GFP antibodies and anti-FLAG antibodies, which did not include antibodies against the His-tag.

## 4. Discussion

Polyclonal antibodies produced in rabbits are widely used in research. Compared to monoclonal antibodies they are easier to produce and are also cheaper. Monoclonal antibodies are specific for only one epitope of the antigen that was used for immunization, and the chance that they recognize unspecific targets is rather low [4]. Polyclonal antibodies, on the other hand, might also contain antibodies which could recognize unspecific targets. This possibility is particularly a problem with regard to antibodies produced against proteins that have been expressed in *E. coli*. These proteins are often expressed as fusion proteins, which is sometimes the only possibility to obtain certain proteins for immunization. The fusion partners often function to increase the solubility of the target protein but may also be used as affinity tags for purification. The widely used His-tag, for instance, allows fusion proteins to be purified by immobilized metal ion affinity chromatography [7]. However, it was found that *E. coli* contains proteins with consecutive histidine residues, which will be co-purified by immobilized metal affinity chromatography [15], especially if the expression level of the fusion protein is low. An *E. coli* strain has been bred with a reduced level of contaminating proteins [16], but this cannot totally circumvent the problem. If fusion proteins are only purified with a single step of immobilized metal affinity chromatography, this leads to contaminations with other bacterial proteins. Antibodies produced against such fusion proteins will therefore be contaminated with antibodies against bacterial proteins. In addition, there will also be some antibodies against other *E. coli* proteins. It will therefore be important to purify these polyclonal antisera to remove unspecific antibodies against both the *E. coli* proteins and the fusion partner.

A method has been proposed to remove antibodies against *E. coli* proteins and also against fusion partners from antisera produced against fusion proteins expressed in *E. coli*. This negative purification method should remove antibodies that are not directed against the protein of interest [8]. For that, the fusion without the protein of interest (empty vector) is also expressed in *E. coli*. Bacteria are lysed, and proteins are bound to nitrocellulose membranes, which are then incubated with the antiserum. After that, the antiserum should theoretically contain only antibodies against the target protein. To completely remove all unspecific antibodies might require several rounds of purification, but this will also lead to a reduction in the level of specific antibodies. In this method, it is also extremely important that the nitrocellulose membrane is completely saturated. Otherwise, unspecific binding of antibodies might occur.

We propose here to use instead positive selection to obtain antibodies specific to the target protein from antisera. Using antiserum obtained against a fusion protein containing His-tag, FLAG-tag, and GFP, we purified pure antibodies against GFP and FLAG-tag, respectively. To isolate GFP-specific antibodies we expressed GFP without a tag in *E. coli*. The expression level of GFP was very high, and it could easily be purified by anion exchange chromatography, ammonium sulfate precipitation, and organic extraction [10] and coupled to an NHS column. As demonstrated, the antibodies purified with this GFP were highly specific. However, it will not always be possible to easily obtain the target protein in high purity for affinity chromatography. If the target protein is easily purified in enough quantity, one might also use that directly for immunization. 

If the protein for affinity chromatography is not easily obtained, it should be expressed with a different fusion tag in a different *E. coli* compartment than the fusion protein used as antigen. We used CusF as fusion for the FLAG-tag and expressed it in the periplasm with the help of its native signal peptide. CusF::FLAG was purified in a single step from the periplasmatic fraction using copper ion affinity chromatography. The rational of expression in the periplasm is that it contains a different set of proteins than the cytoplasm. Thus, if the antiserum is contaminated with antibodies against cytoplasmatic proteins, these contaminating antibodies would not be captured by the fusion protein expressed in the periplasm even if it is not highly purified. Of course, care should be taken when purifying the periplasmatic fraction to not contaminate it with cytoplasmatic proteins. In addition, using different fusion tags for the antigen and for the protein used for affinity purification results in an antibody fraction without antibodies against the fusion tag used for antigen production.

Two weak bands appeared in fraction A1 (Figure 2). These were sometimes detected by the anti-FLAG antibody (Figure 2 and Figure 3), probably depending on the amount of protein loaded on the gel and also the time of reaction with the antibody and the exposure time of the Western blot. They could be a dimer and a N-terminal deletion of the CusF::FLAG protein. Processing is also found in the extract of the antigen construct which leads to smaller bands that are recognized by both antibodies (Figure 3). Again, there is also a weak larger band which could be a dimer because it is recognized by both antibodies. Thus, both antibodies do not react with any unspecific *E. coli* protein.

Agaton et al. [17] have also shown that coupling fusion proteins to NHS-activated sepharose leads to a mixture of target-specific antibodies and antibodies specific for the fusion partner or affinity tag, respectively. They used a stepwise purification against the affinity tag and fusion partner and then against the target protein with affinity tag to obtain target specific antibodies. However, they did not exclude the affinity tag from the final purification step, and therefore, unwanted co-purification of tag-specific antibodies cannot completely be excluded. By using different fusion partners and expression in different compartments, we were able to obtain highly specific antibodies in a single affinity chromatographic step. 

Affinity capture has also been used with peptide fragments to isolate monospecific antibodies that are similar to monoclonal antibodies in that they recognize only one epitope of a protein [18]. The authors coupled peptide fragments representing different parts of the antigen used for immunization to a matrix, which was then used to isolate the corresponding monospecific antibodies. They showed that while the polyclonal antibody recognized the antigen on Western blots, in immunohistochemistry and in immunofluorescence, the monospecific antibodies were mostly only useful for one application, if at all. Thus, this method requires much work to characterize the obtained monospecific antibodies. In many cases, such monospecific antibodies would not be necessary, and a purified polyclonal antibody would be fully satisfactory.

## 5. Conclusions

We present a method to purify polyclonal antibodies produced against fusion proteins. The target protein was expressed with different tags and in different *E. coli* compartments for antibody production and affinity chromatography. This allowed us to purify the antibodies against the target protein in a single chromatographic step. If the target protein can be expressed without tag and purified, it may also be used for the affinity chromatography.

## Supplementary Materials

The following are available online at https://www.mdpi.com/2409-9279/3/2/37/s1, Table S1: Primers used, Figures S1–S6: Sequences of the constructs.

## Abbreviations

GFP	Green Fluorescent Protein;
CBD	Cellulose Binding Domain;
TRX	Thioredoxin,
CAP	Cysteine-rich secretory proteins, antigen 5, and pathogenesis-related protein 1,
CV	Column Volume
