# Supplementary material for: Positive Selection of Specific Antibodies Produced against Fusion Proteins
Source: Methods Protoc. 2020 May 8;3(2):37. doi: 10.3390/mps3020037 (PMC7359703; doi:10.3390/mps3020037)
Supplement: Supplementary file 1 [file mps-03-00037-s001.pdf]

Supplement

Positive Selection of Specific Antibodies Produced against Fusion Proteins

Lukas Kramberger-Kaplan[1], Tina Austerlitz[1] and Holger Bohlmann[1]*

[1] Institute of Plant Protection, Department of Crop Sciences, University of Natural Resources and Life Sciences, Vienna, Austria.

*Corresponding author

Table S1: Primers used in this work (restriction sites are underlined)

oxGFPHisFlagBamrev	TAAGGATCCTCATTTGTCGTCATCGTCTTTGTAGTCACTGCCAGATCCGTGATGGTGATGGTGATGAGATCCGCCAGCACCTTTG	Cloning antigen construct
FlagHisoxGFPNdefor	TTACATATGGACTACAAAGACGATGACGACAAAGGAGGTGCTTCTCATCACCATCACCATCACGGATCTGGCAGTGTTTCTAAAGCTGAAGAAC	
sfGFPforNde	TATCATATGGTTTCTAAAGGTGAAGAACTGTTC	Clone oxGFP without tags
FLOURrevBam	TTCGGATCCTTAAGATCCGCCAGCACC	
CusFSPforNde	AGTCAGTCACATATGAAAAAAGCACTGCAAGTC	Amplify CusF including SP and introducing linker with TEV site
CusFTEVrevBam	TTTGGATCCTTACTGGAAGTACAAGTTTTCAGAACCACTGCCAGATCCCTGGCTGACTTTAATATC	
GS3FLAGrevBam	TAAGGATCCTCATTTGTCGTCATCGTCTTTGTAGTCAGAACCACTGCCAGATCC	Attach FLAG tag to CusF and CBD
CBDCEXforNde	AAACATATGGCCTCTGGTCCGGCTGG	Cloning CBD::FLAG
CAPrevBam	aaaGGAtcCtTTAGTAAGGCCACTCGCCAAC	Amplify CAP (At4g33720) with N-terminal TEV site
TEVCAPfor	aaCTtGtaCttCcagcaagacagtcctcaag	

MDYKDDDDKGGASHHHHHHGSGSVSKAEELFTGVVPILVELDGDVNGHKFSVRGEGEGDATN
GKLTLKFISTTGKLPVPWPTLVTTLTYGVQSFSRYPDHMKQHDFFKSAMPEGYVQERTISFK
DDGTYKTRAEVKFEGDTLVNRIELKGIDFKEDGNILGHKLEYNFNSHNVYITADKQKNGIKA
NFKIRHNVEDGSVQLADHYQQNTPIGDGPVLLPDNHYLSTQSVLSKDPNEKRDHMVLLEFVT
AAGITHGMDELYKGAGGSHHHHHHGSGSDYKDDDDK

Fig. S1

Sequence of antigen construct. FLAG-tag yellow, His-tag blue, oxGFP green

MVSKAEELFTGVVPILVELDGDVNGHKFSVRGEGEGDATNGKLTLKFISTTGKLPVPWPTLV
TTLTYGVQSFSRYPDHMKQHDFFKSAMPEGYVQERTISFKDDGTYKTRAEVKFEGDTLVNRI
ELKGIDFKEDGNILGHKLEYNFNSHNVYITADKQKNGIKANFKIRHNVEDGSVQLADHYQQN
TPIGDGPVLLPDNHYLSTQSVLSKDPNEKRDHMVLLEFVTAAGITHGMDELYKGAGGS

Fig. S2

Sequence of oxGFP without tags. oxGFP green

MKKALQVAMFSLFTVIGFNAQA**NEHHHETMSEAQPQVISATGVVKGIDLESKKITIHHDPIA**
AVNWPEMTMRFTITPQTKMSEIKTGDKVAFNFVQQGNLSLLQDIKVSQGSGSGSDYKDDDDK

Fig. S3

Sequence of CusF::FLAG. FLAG-tag yellow, signal peptide grey, CusF bold

MKKALQVAMFSLFTVIGFNAQA**NEHHHETMSEAQPQVISATGVVKGIDLESKKITIHHDPIA**
AVNWPEMTMRFTITPQTKMSEIKTGDKVAFNFVQQGNLSLLQDIKVSQGSGSGSENLYFQQD
SPQDFLAVHNRARAEVGVGPLRWDEKVAAYARNYANQRKGDCAMKHSSGSYGENIAWSSGSM
TGVAAVDMWVDEQFDYDYDSNTCAWDKQCGHYTQVVWRNSERLGCAKVRCNNGQTFITCNYD
PPGNWVGEWPY

Fig. S4

Sequence of CusF::CAP. CusF bold, signal peptide grey, TEV site pink

MASGPAGCQVLWGVNQWNTGFTANVTVKNTSSAPVDGWTLTFSFPSGQQVTQAWSSTVTQSG SAVTVRNAPWNGSIPAGGTAQFGFNGSHTGTNAAPTAFSLNGTPCTVGTPTPTPTTGSGSDYKDDDDK

Fig. S5

Sequence of CBD::FLAG. CBD-CEX bold, FLAG-tag yellow

MKHHHHHH**PMSDKIIHLTDDSFDTDVLKADGAILVDFWAEWCGPCKMIAPILDEIADEYQGK LTVAKLNIDQNPGTAPKYGIRGIPTLLLFKNGEVAATKVGALSKGQLKEFLDANLA**GSGSGS ENLYFQ

Fig. S6

Sequence of His::TRX. His-tag blue, TRX bold, TEV site pink
